# The influence of media use degree on public depressive symptoms: mediating role of big five personality

**DOI:** 10.1186/s12888-023-05097-w

**Published:** 2023-08-22

**Authors:** Fangmin Gong, Yuhan Jia, Xinying Sun, Hewei Min, Xiaocen Jia, Fei Wang, Xincheng Huang, Xin Lin, Zheming Li, Yibo Wu

**Affiliations:** 1 School of Literature and Journalism Communication, Jishou University, Jishou, China; 2 School of Public Health, Peking University, Beijing, China; 3 School of Public Health, Qingdao University, Qingdao, China; 4 State Key Laboratory of Cognitive Neuroscience and Learning, Beijing Normal University, Beijing, China; 5 School of Economics and Management, Beijing Institute of Graphic Communication, Beijing, China; 6 Department of Stomatology, North Sichuan Medical College, Sichaun, China; 7 School of basic medicine, Peking University Health Science Center, Beijing, China; 8 Xiangxi Tujia and Miao Autonomous Prefecture, 120 Renmin South Road, Jishou City, Hunan Province China; 9 38 Xueyuan Road, Haidian District, Beijing, China

**Keywords:** Media use, Big five personality, Depressive symptoms, Mediation model, Regression analysis

## Abstract

**Background:**

Mixed results have been found regarding the relationship between media use degree and depressive symptoms. The purpose of this study is to explore the relationship between media use degree, big five personality and depressive symptoms with a mediation model.

**Method:**

This was a cross-sectional study. With 9-item Patient Health Questionnaire (PHQ-9), 10-item Big Five Inventory (BFI-10) and self-designed media usage scale, 11,031 participants aged 12 and above in 120 cities in China were collected. Pearson correlation analysis and regression analysis were performed on the data. The Process plug-in was used to construct the mediation model and explore the relationship among media use degree, big five personality and depressive symptoms. The nonparametric percentile Bootstrap method was used to test the mediating effect of personality traits.

**Results:**

The degree of media use was positively correlated with depressive symptoms (*r* = 0.20, *P <* 0.001), and big five personality played a mediating role between the degree of media use and depressive symptoms. Among five traits, extroversion (*r*=-0.12, *P <* 0.001), conscientiousness (*r*=-0.23, *P <* 0.001), openness (*r*=-0.03, *P <* 0.01) and agreeableness (*r*=-0.22, *P <* 0.001) were negatively correlated with depressive symptoms, and neuroticism (*r* = 0.25, *P <* 0.001) were positively correlated with depressive symptoms. In addition, extraversion (-0.004, -0.001), conscientiousness (-0.015, -0.008), agreeableness (-0.008, -0.001) and neuroticism (-0.015, -0.007) in big five personality played a mediating role between media use and depressive symptoms.

**Conclusion:**

The degree of media use positively predicted depressive symptoms, and excessive media use may bring risks to mental health. People with high neuroticism, low agreeableness, low conscientiousness and low extroversion are more likely to suffer from depressive symptoms.

## Introduction

Depression is one of the most common mental disorders. Depression is a kind of emotion, which refers to the psychological state that individuals feel constrained and depressed. It is a typical negative emotional experience and can develop into depression in severe cases [1]. Depression has become one of the main causes of people’s disability and premature death [2]. Depression accounts for more disability-adjusted life years than all other mental disorders [3]. More than 300 million people worldwide suffer from depression [4]. By 2030, depression will become the largest disease burden in the world [5]. In many countries, the prevalence of depression is increasing significantly [6–8], especially during the COVID-19 epidemic [9]. Such as in China, Zhong found that the government’s blockade and control of Wuhan in the early stage of the epidemic led to many people’s depression [10]., and frontline healthcare workers showed the greatest severity of distress symptoms [11]. The incidence of depression in isolated people was higher during the epidemic period [12]. The confirmed and suspected infected people and frontline workers are more prone to depression, and the incidence of suicidal ideation is higher [13]. At the same time, the COVID-19 epidemic has also caused people to spend more time in the media, seeking mental health problems [14]. Therefore, it is necessary to explore the mechanism of public depression, so as to provide theoretical support for its prevention and corresponding intervention strategies.

Many scholars have studied the risk factors of depression, such as low social support [15] and high life pressure [16]. As the rapid increase of media use, people are increasingly worried that the interaction with media may lead to the impairment of psychological and social functions [17]. Many studies have shown that media use, such as social media, video games, TV and movies was related to the development of public depression [18–22].

Media refers to the tools or carriers that people use to transmit and obtain information, including traditional media (traditional mass communication mode, media that regularly release information to the public or provide educational and entertainment platforms through a certain platform, such as newspapers, magazines, radio, television, etc.) and new media (using digital technology to provide users with information and services through computer networks, wireless communication networks, satellites and other channels, such as computers and mobile phones, etc.). The increase of media use function will lead to the decreased top-down attentional control associated with media multitasking [23] could disrupt active coping mechanisms that promote the rapid shift of attention away from negative stimuli [24], thereby resulting in heightened depression [25]. A group study showed that more use of the Internet would lead to less communication between participants and family members, smaller social circle and more depression and loneliness [26]. The higher the degree of internet use, the more clinical symptoms of severe depression appear with the increase of online time [27]. A study of Finns aged 12–18 found that depressed teenagers use mobile phones more than non-depressed teenagers [28]. In conclusion, the degree of media use may affect public depression. Accordingly, we propose hypothesis H1: The degree of media use can positively predict public depression.

Personality psychology points out that personality traits can distinguish stable differences among different individuals and predict behavior tendency. Personality can be characterized by a series of five dimensions, namely Big Five Personality Model [29]. According to the Big Five Personality Model, the constitution of personality has five dimensions: Conscientiousness, Extraversion, Agreeableness, Openness and Neuroticism. By summarizing the research of scholars such as Brown [30] and Kendler [31], Steunenberg and others put forward that there is an established relationship between personality characteristics and depression, and the most complete etiological model of depression symptoms may need to include all three types of influencing factors: personality, health-related and social situational factors [32]. Individuals with different personality traits will show different personality tendencies. Studies have shown that extroversion, agreeableness, conscientiousness and openness of Big Five personality were negatively correlated with depressive symptoms, while neuroticism was positively correlated with depressive symptoms [33]. There was also some evidence that depression was related to lower levels of extroversion, agreeableness and neuroticism, and to a lesser extent, it was related to conscientiousness and openness [34]. In depression, neuroticism is the most common and the most serious [35]. Neuroticism is a personality structure characterized by emotional response, anxiety and negative emotions [36], which is widely considered to be related to the high risk of depression [37, 38].

According to the cognitive affective personality system (CAPS) theory, the personality state changes [39]. Personality state is the activation mode of cognition and emotion in personality system at a certain moment, which has variability [40]. With its powerful function, the media changes the public’s cognition and plays the role of activator at some important moments. The utility and function of mass media is an important part of people’s personality formation [41]. The use of media can exert a subtle influence on personality [42].

The degree of media use has different effects on depression due to personality factors [43]. Some scholars found that there was a positive correlation between media use and mental health [44]. Some scholars even thought that media use had both positive and negative effects on depression [45]. Besides, some scholars have found that there was no relationship between media use and depression [46]. The results of whether the degree of media use leads to depression are mixed, and no clear conclusion can be drawn, so further research is needed [47–49]. Shakya and Christakis found that the use of Facebook was related to the low level of subjective well-being, but unfortunately the role of personality traits in it was not verified [50]. Therefore, it is particularly important to clarify what role Big Five personality plays between the degree of media use and public depression.

To sum up, it is necessary to explore the mediating role of the five personality types of Big Five personality in the degree of media use and depression. This study puts forward the hypothesis H2: Big Five personality plays an intermediary role between the degree of media use and depression. H2a: Extroversion plays an intermediary role between the degree of media use and depression. H2b: Conscientiousness plays an intermediary role between the degree of media use and depression. H2c: Agreeableness plays an intermediary role between the degree of media use and depression. H2d: Openness plays an intermediary role between the degree of media use and depression. H2e: Neuroticism plays an intermediary role between the degree of media use and depression.

Some studies have found that individuals of different genders have different possibilities of depression. In essence, women are more likely to suffer from depression, and they are also more susceptible to some significant social factors that induce or activate depression [51]. Women have more incidence of depression than men in the whole life cycle or at the beginning of puberty, which is related to the fact that women experience or produce more stressors at the beginning of puberty [52]. Scarinci found that young women were more likely to show depressive symptoms than older women [53]. The higher the education level of women, the less likely they were to show depressive symptoms. The influence of education level on depression is not limited to women. Bjelland found that, for both men and women, higher education level plays a protective role in lifelong depression, but men have stronger correlation in the relationship between education level and depression [54]. The level of education and income represent the level of people’s social and economic status. Higher social and economic status, that is, good educational background, higher income and family property, is conducive to reducing the degree of depression [55]. Therefore, we also made an exploratory analysis to test the controlling effect of age, education level and family economic status.

**Fig. 1:**
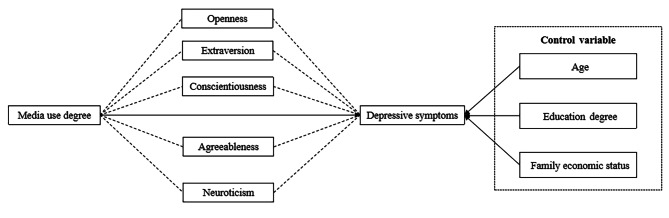
The theoretical model hypothesis diagram

Individuals are in the era of media, and ubiquitous media information contact plays an important role in influencing public depression. Depression is a process of complex interaction between individuals and many social factors, which is intertwined with media information ecology and personality traits. Therefore, this study takes 11,031 people in China as the research object, combines the hypothesis theory of media information ecology and personality traits, takes age, education level and family economic status as the control variables, explores the mechanism path that the degree of media use affects depression. By constructing an intermediary model (Fig. 1), the mediating role of five personality types between the degree of media use and depressive symptoms was investigated.

## Methods

### Data collection

This survey was conducted by multistage sampling from July 10th, 2021 to September 15th, 2021. A total of 23 provinces, 5 autonomous regions and 4 municipalities (Beijing, Tianjin, Shanghai and Chongqing) in China were included. Using the random number table method, 2–6 cities are selected from the non-provincial administrative regions of each province and autonomous region, totaling 120 cities. Next, openly recruit investigators or investigation teams (consisting of ≤ 10 people) in these cities. Based on the data of the “Results of the Seventh National Census in 2021”, 120 urban residents were sampled by quota (the quota attributes are gender, age and urban-rural distribution), so that the gender, age and urban-rural distribution of the samples obtained basically conform to the demographic characteristics. Finally, with the help of the Internet Questionnaires Platform (https://www.wjx.cn/), the investigators distributed online questionnaires to the public face to face and one to one in their respective regions, and the respondents answered them by clicking on the link. The informed consent of the subjects was obtained during the investigation, and the questionnaire number was input by the investigator. If the respondent has thinking ability but not enough action ability to answer the questionnaire, the investigator will conduct one-on-one inquiry and answer it instead.

### Outcome measures

In this study, the Patient Health Questionnaire (PHQ-9) was used to evaluate depressive symptoms [56]. The PHQ-9 was previously translated into Chinese and validated by researchers in China [57, 58]. There were 9 items in the scale, including four options: Not at all, Several days, More than half the days, and Nearly every day. The scale was assigned 0–3 in turn (Not at all = 0, Nearly every day = 3), and the score was between 0 and 27. The scale conforms to DSM-V standard and is used to identify depression. The higher the score of the respondents, the more serious the depressive symptoms were. The Cronbach’s alpha of the PHQ-9 was 0.94. Through KMO and spherical Bartlett test, KMO statistic was 0.88, and spherical test *P* < 0.01, which indicated that factor analysis was appropriate. Two factors were extracted from nine variables (items), and the total variance of the two factors was 52.38%[59].

A 7-item self-made scale was used to measure the frequency of media use. In order to know how the participants use the media, the members of the research team consulted books and literature scientifically and comprehensively, and then designed a questionnaire to ensure that the questionnaire was suitable for measuring the media use of all people [60]. There were 7 items in the scale, which respectively know the contact frequency of respondents to seven kinds of media: newspapers, magazines, radio, television, books (non-textbooks), personal computers (including tablets) and smart phones. The scale has five options: Never use, Occasionally use (≤ 1 day/week), Sometimes use (2 ~ 3 days/week), Frequently use (4 ~ 5 days/week) and Almost every day (6 ~ 7 days/week), which were assigned to 1–5 in turn (Never use = 1, Almost every day = 5). The number of days that the measured person used various media in one week was used as the scoring basis, and the total score of each option was added as the scoring result, with a total score of 35 points. The higher the score, the more frequently the measured person uses the media. The Cronbach’s alpha of the scale was 0.70. Through KMO and spherical Bartlett test, KMO statistic was 0.74, and spherical test *P* < 0.01, which indicated that factor analysis was appropriate. Two factors were extracted from seven variables (items), and the total variance of the two factors was 61.80%. Confirmatory factor analysis of the convergence validity of the media use scale showed that CMIN/DF = 2.44 and RMSEA (root mean square error) was within the range of < 0.05. The test results of GFI, TLI, IFI and CFI all reached the excellent level of above 0.9. The convergence validity (AVE) and combination reliability (CR) of each dimension of the scale were tested. The results showed that the standard load range of the scale items was higher than 0.5 from 0.57 to 0.87, the value of AVE is 0.52 higher than 0.5, and the value of CR is 0.88 higher than 0.7. Therefore, the scale used in this study has good convergence validity and combination reliability.

The 10-item Big Five Inventory (BFI-10) was used to assess personality [61]. The scale consists of 10 items, which measure five factors of personality, namely extraversion, conscientiousness, openness, agreeableness and neuroticism. Ten items include five options: Strongly disagree, a little disagree, neither agree nor disagree, a little agree and very agree. The scores of these five options are 0–4 from strongly disagree to strongly agree. Among them, 5 items were scored positively and 5 items are scored reversely. The higher the score of each dimension, the more obvious the personality trait is. Through KMO and spherical Bartlett test, KMO statistic was 0.68, and spherical test *P* < 0.01, which indicated that factor analysis was appropriate. The factor load was 0.41 ~ 0.97, discriminant correlations for the BFI-10 were all < 0.4, the validity of the scale was acceptable [62].

### Statistical analyses

SPSS 26.0 was used for statistical analysis. The data that do not obey the normal distribution were represented by M (QL, QU), and chi-square test was used for comparison between groups. Pearson correlation analysis and regression analysis were carried out, and nonparametric percentile Bootstrap method was used for model construction and intermediary effect test. Using Process plug-in V2.16.3 to verify the mediation model, the 95% confidence interval of the mediation effect was estimated by extracting 5000 samples from the original samples. If the confidence interval did not contain 0, it indicates that the intermediary effect exists. otherwise, the intermediary effect did not exist, α = 0.05.

## Results

### Descriptive statistics and one-way ANOVA

A total of 11,031 valid questionnaires were collected in this study. Among them, 5998 were women and 5033 were men. 1065 people aged ≤ 18 years, 5332 people aged 19–40 years, 3795 people aged 41–65 years and 875 people aged ≥ 66 years (According to the age of minors [63], young people [64], middle-aged and old people [65]). There were 1,127 people with primary school education or below, 1,439 people with junior high school education, 1,978 people with secondary school education or high school education, 5,750 people with college education or above, and 737 people with master’s and doctoral degrees. There were 3,246 families with a monthly income of ≤ 3,000, 5,325 families with a monthly income of 3,001–7,500, and 2,460 families with a monthly income of ≥ 7,501 (Table 1).

The differences of depressive symptoms were statistically significant (P < 0.001) in gender, age, education level and family economic status, indicating that these factors have a significant impact on residents’ depressive symptoms.

**Table 1:** Socio-demographic characteristics (N = 11,031)

Variable	Category	Number of people	Percentage (%)	*χ2*	*P*
Gender				110.90	＜0.001
	Male	5033	45.6		
	Female	5998	54.4		
Age/years				193.58	＜0.001
	≤ 18	1065	9.7		
	19–40	5332	48.3		
	41–65	3759	34.1		
	≥ 66	875	7.9		
Education degree				181.36	＜0.001
	Primary school and below	1127	10.2		
	Middle School	1439	13.1		
	Technical secondary school or high school	1978	17.9		
	Junior college or university degree	5750	52.1		
	Master or doctoral candidate	737	6.7		
Family economic status				105.14	＜0.001
	≤ 3000	3246	29.4		
	3001–7500	5325	48.3		
	≥ 7501	2460	22.3		

### Common method bias

As for statistical control, this study adopted Harman single factor test, and made exploratory factor analysis on all items of research variables. The results showed that the eigenvalues of six factors were greater than 1, and the variance explained by the first factor is 25.71%, which was less than the critical standard of 40%. Therefore, it can be concluded that there was no serious common methodological deviation in this study.

### Scores on media use, big five personality, depressive symptoms among participants

The scores of all scales of the included people were shown in Table 2. The total score of the media use scale was 19(16,22), newspapers, magazines and broadcast had similar scores, and smartphones scored the highest, with 5(4,5). It showed that Chinese citizens were more inclined to smart phones in media use. The subjects’ scores of big five personality Scale were similar, with the highest score of 7(6,8) for agreeableness and the lowest score of6(5,6) for neuroticism. The subjects’ total score of depressed symptoms scale was 5(1,9).

**Table 2:** Scores of several scales among subjects (N = 11,031)

Scales	Items	Range of scores	M ± SD	M(QL,QU)	*Z*	*P*
**Media use**	7	7–35	19.34 ± 4.96	19(16,22)	0.08	< 0.001
Newspaper	1	1–5	1.86 ± 1.08	1(1,2)	0.29	< 0.001
Magazine	1	1–5	1.91 ± 1.05	1(1,2)	0.26	< 0.001
Book (non-textbook)	1	1–5	2.73 ± 1.26	3(2,4)	0.17	< 0.001
Broadcast	1	1–5	2.10 ± 1.19	1(1,2)	0.25	< 0.001
TV	1	1–5	3.24 ± 1.28	3(2,4)	0.16	< 0.001
PC (including tablet)	1	1–5	3.17 ± 1.44	3(2,4)	0.17	< 0.001
Smartphone	1	1–5	4.33 ± 1.13	5(4,5)	0.39	< 0.001
**Big five personality**						
Extroversion	2	2–10	6.29 ± 1.61	6(5,7)	0.20	< 0.001
Conscientiousness	2	2–10	6.84 ± 1.61	6(6,8)	0.20	< 0.001
Openness	2	2–10	6.47 ± 1.54	6(6,7)	0.22	< 0.001
Agreeableness	2	2–10	7.00 ± 1.49	7(6,8)	0.19	< 0.001
Neuroticism	2	2–10	5.74 ± 1.50	6(5,6)	0.22	< 0.001
**Depressive symptoms**	9	0–27	6.18 ± 5.68	5(1,9)	0.26	< 0.001
Little interest or pleasure in doing things	1	0–3	0.82 ± 0.77	1(0,1)	0.26	< 0.001
Feeling down, depressed, or hopeless	1	0–3	0.66 ± 0.75	1(0,1)	0.30	< 0.001
Trouble falling or staying asleep, or sleeping too much	1	0–3	0.82 ± 0.83	1(0,1)	0.24	< 0.001
Feeling tired or having little energy	1	0–3	0.87 ± 0.79	1(0,1)	0.27	< 0.001
Poor appetite or overeating	1	0–3	0.72 ± 0.75	1(0,1)	0.27	< 0.001
Feeling bad about yourself—or that you are a failure or have let yourself or your family down	1	0–3	0.66 ± 0.78	0(0,1)	0.30	< 0.001
Trouble concentrating on things, such as reading the newspaper or watching television	1	0–3	0.69 ± 0.79	1(0,1)	0.29	< 0.001
Moving or speaking so slowly that other people could have noticed? Or the opposite—being so fidgety or restless that you have been moving around a lot more than usual	1	0–3	0.57 ± 0.77	0(0,1)	0.35	< 0.001
Thoughts that you would be better off dead or of hurting yourself in some way	1	0–3	0.37 ± 0.72	0(0,1)	0.45	< 0.001

### Relationship between media use, big five personality and depressive symptoms

Table 3 showed the correlation among media use degree, big five personality and depressive symptoms. The results showed that media use degree (*r* = 0.20, *P* < 0.001) was positively correlated with depressive symptoms. Media use degree was positively correlated with extraversion (*r* = 0.03, *P* < 0.01), conscientiousness (*r* = 0.04, *P* < 0.001), openness (*r* = 0.15, *P* < 0.001), agreeableness (*r* = 0.04, *P* < 0.001) of the big five personality Scale,and negatively correlated with neuroticism (r=-0.05, P < 0.001). Among the big five personality, extraversion (*r*=-0.12, *P* < 0.001), conscientiousness (*r*=-0.23, *P* < 0.001), openness (*r*=-0.03, *P* < 0.01), agreeableness (*r*=-0.22, *P* < 0.001) were negatively correlated with depressive symptoms, and positively correlated with neuroticism (*r* = 0.25, *P* < 0.001). In addition, age (*r*=-0.07, *P* < 0.001) and family economic status (*r*=-0.05, *P* < 0.001) were negatively correlated with depressive symptoms, while education degree (*r* = 0.05, *P* < 0.001) was positively correlated with depressive symptoms.

**Table 3:** Correlation analysis of related factors of depressive symptoms (*r*)

Variable	Age/years	Education degree	Family economic status	Media use degree	Extraversion	Conscientiousness	Openness	Agreeableness	Neuroticism	Depressive symptoms
Age/years	1									
Education degree	-0.32^***^	1								
Family economic status	-0.04^***^	0.29^***^	1							
Media use degree	-0.12^***^	0.32^***^	0.16^***^	1						
Extraversion	-0.04^***^	-0.03^***^	0.08^***^	0.03^**^	1					
Conscientiousness	0.25^***^	-0.07^***^	0.04^***^	0.04^***^	0.18^***^	1				
Openness	-0.21^***^	0.18^***^	0.09^***^	0.15^***^	0.19^***^	0.07^***^	1			
Agreeableness	0.02^*^	0.06^***^	0.03^***^	0.04^***^	-0.01	0.29^***^	0.14^***^	1		
Neuroticism	-0.05^***^	0.02	-0.07^***^	-0.05^***^	-0.18	-0.19^***^	-0.08^***^	-0.28^***^	1	
Depressive symptoms	-0.07^***^	0.05^***^	-0.05^***^	0.19^***^	-0.12^***^	-0.29^***^	-0.03^***^	-0.22^***^	0.25^***^	1

^***^P＜0.001,^**^P＜0.01,^*^P＜0.05

### Hierarchical regression analysis of predictive variables on depressive symptoms

In order to further explore the influence of each predictor on personal depressive symptoms, this study conducted hierarchical regression analysis (Table 4). Firstly, the age, education level and family economic status were put into the equation. Age (*b*=-0.06, *P* < 0.001), education degree (*b* = 0.05, *P* < 0.001) and average household monthly income (*b*=-0.07, *P* < 0.001) had significant influence on depressive symptoms. R^2^ was 0.01. Secondly, media use was the second factor in the equation. Media use degree (*b* = 0.21, *P* < 0.001) had become a significant influencing factor of personal depressive symptoms, which showed that the deeper personal media use, the more likely it was to be depressed. At this time, R^2^ increased from 0.01 to 0.05. Finally, the five dimensions of big five personality, an intermediary variable, were introduced into the model. At this time, the influence of media use degree on depressive symptoms was still statistically significant, and the partial regression coefficient increased from 0.21 to 0.24. The extraversion, conscientiousness, agreeableness and neuroticism in the big five personalities had statistical significance on depressive symptoms. R^2^ increased from 0.05 to 0.16. Among them, extraversion (*b*=-0.23, *P* < 0.001), conscientiousness (*b*=-0.53, *P* < 0.001), agreeableness (*b*=-0.52, *P* < 0.001) and depressive symptoms were negative predictors, neuroticism (*b* = 0.66, *P* < 0.001) had a positive predictive effect on depressive symptoms, openness (*b*=-0.01, *P >* 0.05) had nothing to do with depressive symptoms. The results showed that big five personality and media use degree were the influencing factors of depressive symptoms.

**Table 4:** Hierarchical regression analysis of depressive symptoms (*b*)

Forecast variable	model 1			model 2			model 3		
	B(SE)	*Beat*	*t*	B(SE)	*Beat*	*t*	B(SE)	*Beat*	*t*
Age/years	-0.44 (0.07)	-0.06	-5.90^***^	-0.41 (0.07)	-0.06	-5.59^***^	-0.06 (0.07)	-0.01	-0.81
Education degree	0.26 (0.05)	0.05	4.78^***^	-0.05 (0.06)	-0.01	-0.97	-0.10 (0.05)	-0.02	-1. 86
Family economic status	-0.54 (0.08)	-0.07	-6.88^***^	-0.67 (0.08)	-0.09	-8.69^***^	-0.45 (0.07)	-0.06	-6.16^***^
Media use degree				0.24 (0.01)	0.21	21.43^***^	0.27 (0.01)	0.24	25.40^***^
Extraversion							-0.23 (0.03)	-0.07	-7.09^***^
Conscientiousness							-0.53 (0.03)	-0.15	-15.39^***^
Openness							-0.01 (0.03)	-0.01	-0.20
Agreeableness							-0.52 (0.04)	-0.14	-14.31^***^
Neuroticism							0.66 (0.04)	0.18	18.81^***^
F	38.16			144.61			240.79		
R^2^	0.01			0.05			0.16		

^***^P＜0.001,^**^P＜0.01,^*^P＜0.05

### Mediation analysis of big five personality

**Fig. 2:**
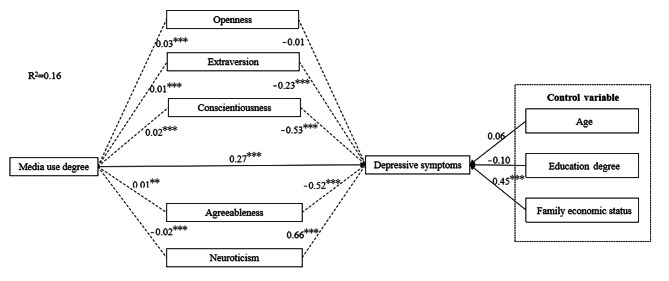
The intermediary verification model of big five personality. ^***^P＜0.001,^**^P＜0.01,^*^P＜0.05

According to the regression results and research hypothesis, this study conducted an intermediary effect test, in order to clarify the mechanism that the degree of media use affects personal depressive symptoms. The Fig. 2 showed that media use was significantly related to the five dimensions of big five personality. Consistent with the results of hierarchical regression, neuroticism (*b* = 0.66, *P* < 0.001) in big five personality had a significant positive impact on personal depressive symptoms, extroversion (*b*=-0.23, *P* < 0.001), conscientiousness (*b*=-0.53, *P* < 0.001) and agreeableness (*b*=-0.52, *P* < 0.001) had a significant negative impact on personal depressive symptoms.

After in-depth analysis of the results of intermediary effect test, it is found that after controlling the three variables of age, education level and family economic status, the direct effect of media use degree on depressive symptoms was [Effect = 0.27, 95%CI (0.25,0.29)], which indicates that the deeper the use of media, the easier it was to have depressive symptoms (Table 5). And the indirect effect of media use degree on depressive symptoms was [Effect=-0.03, 95%CI (-0.04, -0.03)], it shows that Big Five personality has a mediating effect in the relationship between the degree of media use and depressive symptoms. Among them, the confidence interval of extroversion, conscientiousness, agreeableness and neuroticism of Big Five personality did not contain 0, which played an intermediary effect between the degree of media use and depressive symptoms.

**Table 5:** Path coefficient of mediating effect model

Path	Effect value	SE	*t*	*P*	95% confidence interval	
					Upper	Lower
Direct effect						
Media use degree→ Depressive symptoms	0.27	0.01	25.40	＜0.001	0.248	0.291
Total indirect effect					-0.037	-0.021
Media use degree→ Extraversion→ Depressive symptoms					-0.004	-0.001
Media use degree→ Conscientiousness→ Depressive symptoms					-0.015	-0.008
Media use degree→ Agreeableness→ Depressive symptoms					-0.008	-0.001
Media use degree→ Openness→ Depressive symptoms					-0.002	0.002
Media use degree→ Neuroticism→ Depressive symptoms					-0.015	-0.007

## Discussion

This study showed that there was a significant positive correlation between the degree of media use and depressive symptoms, and the deeper the degree of media use, the easier it was to be depressed. Assume H1 holds, which was consistent with the existing research results [22]. The use of media may promote the development of depression by strengthening the cognition of depression [66]. Public exposure to different media content may lead to depression [67]. In particular, the frequent occurrence of some negative contents such as bullying and harassment has a negative impact on people’s psychology. In the process of communication, members of online community with depression may be exposed to other members’ negative emotions, thus causing a significant increase in anxiety, anger and other negative emotions, and even the risk of suicide [68]. Although some studies have shown that online interaction between depressed patients and other users can help to relieve depressive symptoms [69], but this is only the interaction of positive emotions [70].

Through literature review, Chen found that most studies in western countries support that media use leads to an increase in individual depression, but the study with China as the subjects confirms that media use has a significant negative predictive effect on depression [71]. On the contrary, the conclusion of this paper supports the positive prediction of depressive symptoms by media use. It may be due to the different intermediary variables used [71]. For example, Yan takes online capital as an mediating variable, and draws the conclusion that the intensity of media use significantly negatively predicts depression [72], while the mediating variable in this study is personality traits.

Although previous studies have shown that media use has an impact on depressed state, the effects of personality traits on media use and depressed state have not been fully studied [73]. We studied whether personality traits play a role between media use and depressive symptoms. This study found that extroversion, conscientiousness, agreeableness and neuroticism in Big Five personality all played a mediating role between the degree of media use and depression. People with high neuroticism, low agreeableness, low conscientiousness and low extroversion are more likely to suffer from depression. Assume H2a, H2b, H2c and H2e hold, which was consistent with the research results of Giota and Kleftaras [74]. People with high neuroticism, low agreeableness, low conscientiousness and low extroversion use media more deeply. Individuals with low extroversion (i.e. introversion) are more likely to use SNS and other technologies to meet their communication needs [75]. Because people with low extroversion and low agreeableness are difficult to establish friendship offline [76]. Butt and Phillips describe that those who were highly neurotic might use the Internet to avoid loneliness [77]. Highly neuroticism individuals prefer to present their ideal and false self on Facebook [78, 79]. Frequent contact with these idealized media images may have a negative impact on the happiness of neuroticism, because they will have more negative emotions after comparing with the unfavorable social reality [80]. Compared with non-neuroticism women, highly neuroticism women show greater dissatisfaction with their bodies after being exposed to the idealized images presented by the media [81]. The media environment full of perfect life pictures may be more threatening to the mental health of highly neurotic people than the offline social world [82, 83].

Research showed that openness had no mediating effect, assuming that H2d was not established. In the past studies, it was often found that there was no significant correlation between openness and depression [84]. Openness is divided into six narrow levels: fantasy, aesthetics, feelings, actions, thoughts and values [85]. However, the relationship between these six dimensions and depression is not the same, only the aesthetic aspect is positively correlated with depression, and the other five aspects are not significant [86]. This may lead to no significant openness and depression. But some scholars believe that there is a positive correlation between openness and depressive symptoms [87]. People with fantasy may suffer from depression, because their ideal state is quite different from their actual state, which is more likely to lead to disappointment and sadness [88]. In future research, researchers need to further explore the relationship between openness and depressive symptoms.

The analysis also showed that people’s age and family economic status were significantly related to media use and depression. The younger the age, the worse the family economic situation, the higher the degree of media use and depression. Chen surveyed 886 junior high school students in three full-time middle schools in Wuhan, and found that excessive use of social networking sites has a significant positive predictive effect on depression [89]. Some studies have shown that three quarters of lifelong mental illness begins before the age of 24[90], and compared with the children of non-floating parents, the risk of depression among left-behind children and adolescents is significantly higher [91]. People with poor economic conditions are more likely to remain in a depressive symptoms, which was consistent with the research results of Chang [92].

### Strengths

Through the analysis of data collected nationwide, this study verified another factor that affects depression-the degree of media use, which has outstanding theoretical value and practical significance. It was found that the degree of media use has a positive effect on depression, and the more frequently media use, the greater the possibility of depression. At the same time, the degree of media use also has the mediating effect of Big Five personalities, and different personalities have different manifestations in the degree of media use and the possibility of depression.

### Limitations

There are some limitations in this research. First of all, people’s media usage only measures the usage days of different media, but can’t fully present the content type and purpose of people’s specific media usage. Secondly, this is a cross-sectional study. Although it is suitable for our research hypothesis, it does not allow us to draw causal conclusions. In the future research, in terms of causal explanation, we can compare people with depression and people without depression through experimental control design, and explore the influence of media on depression. At the same time, this research uses self-report and this is a limit because it increases the risk for social desirability in the responses. Qualitative research methods such as in-depth interviews can be added to further explore the relationship among media, personality and depression, and enhance the persuasiveness of the results.

## Conclusions

This study investigated the relationship between the degree of media use, Big Five personality and depression. The results showed that the degree of media use aggravated people’s depression, and people with high neuroticism, low agreeableness, low conscientiousness and low extroversion were more likely to suffer from depression. The conscious use of media to protect mental health is an important topic in our society. Time spent in the media may pose a risk to a person’s mental health. In order to protect ourselves from the negative effects of media use, it is important to know our personality and how media use affects our mental health. Especially for people with neurotic personality characteristics, they are more likely to suffer from depression.

## Data Availability

Data are available, upon reasonable request, by emailing: wuyibo@bjmu.edu.cn.
